# Size Distribution of Inactivated Tick-Borne Encephalitis Virus Particles Revealed by a Comprehensive Physicochemical Approach

**DOI:** 10.3390/biomedicines10102478

**Published:** 2022-10-04

**Authors:** Andrey V. Moiseenko, Dmitry V. Bagrov, Mikhail F. Vorovitch, Victoria I. Uvarova, Maxim M. Veselov, Anastasia V. Kashchenko, Alla L. Ivanova, Dmitry I. Osolodkin, Alexey M. Egorov, Aydar A. Ishmukhametov, Konstantin V. Shaitan, Olga S. Sokolova

**Affiliations:** 1 Department of Biology, Lomonosov Moscow State University, Moscow 119234, Russia; 2 FSASI “Chumakov FSC R&D IBP RAS” (Institute of Poliomyelitis), Moscow 108819, Russia; 3 Institute of Translational Medicine and Biotechnology, Sechenov First Moscow State Medical University, Moscow 119991, Russia; 4 Department of Chemistry, Lomonosov Moscow State University, Moscow 119234, Russia; 5 Faculty of Biology, MSU-BIT Shenzhen University, 1 International University Park Road, Dayun New Town, Longgang District, Shenzhen 518172, China

**Keywords:** *Flavivirus*, tick-borne encephalitis, electron microscopy, EELS, nanoparticle tracking analysis, cryo-EM

## Abstract

Tick-borne encephalitis virus (TBEV) is an enveloped RNA virus, a member of the genus *Flavivirus* (family *Flaviviridae*). Here, we provide a detailed analysis of the size and structure of the inactivated TBEV vaccine strain Sofjin-Chumakov. Four analytical methods were used to analyze individual TBEV particles—negative staining TEM, cryo-EM, atomic force microscopy (AFM), and nanoparticle tracking analysis (NTA). All methods confirmed that the particles were monodisperse and that their mean size was ~50 nm. Cryo-EM data allowed us to obtain a 3D electron density model of the virus with clearly distinguishable E protein molecules. STEM-EELS analysis detected phosphorus in the particles, which was interpreted as an indicator of RNA presence. Altogether, the described analytical procedures can be valuable for the characterization of inactivated vaccine virus samples.

## 1. Introduction

Flaviviruses (genus *Flavivirus*, family *Flaviviridae*, phylum *Kitrinoviricota*) cause several severe human diseases transmitted by ticks or mosquitoes: yellow fever, dengue fever, West Nile fever, Zika fever, and Japanese and tick-borne encephalitis. Flaviviruses are enveloped viruses: the nucleocapsid is surrounded by a lipid bilayer envelope, where envelope proteins E and M are anchored. The outer protein shell has icosahedral symmetry (T = 3) and consists of 180 copies of the ectodomain of glycoprotein E. The diameter of the viral particle is about 50 nm [1,2]. The 11 kb flavivirus genome encodes three structural proteins (envelope protein E, membrane protein M, and capsid protein C), as well as seven non-structural proteins that form the replication complex [3]. The protein envelope proteins guarantee the stability of the viral particle and mediates the first stages of infection: interaction of the virion with cell receptors, endosome formation, fusion of the viral and cell membranes, and entry of the viral RNA into the cell cytoplasm [4].

Tick-borne encephalitis (TBE) is widely spread in northern Eurasia. The causative agent of the disease is the tick-borne encephalitis virus (TBEV), a typical representative of the genus *Flavivirus*, belonging to the group of tick-borne mammalian flaviviruses [5,6]. In the Russian Federation, more than 61 million people live in areas endemic to TBEV [6,7]. At present, natural foci of TBEV remain highly active; an increase in the abundance of vectors and hosts and an expansion of the area of infection are observed. Two to three thousand cases of the disease, characterized by different degrees of severity, are registered annually.

Preventive vaccination is the primary means of controlling the incidence of TBEV. Five commercial vaccines against TBEV are currently manufactured worldwide, based on Far Eastern or European TBEV subtypes [6,7]. The active component of commercial vaccines is usually formalin-inactivated TBEV virion suspension; thus, commercial vaccines represent the inactivated whole-virion vaccine type. The homogeneity of vaccine preparation was shown to be responsible for the dominance of antibody responses to different sites on protein antigens and can strongly influence the spectrum and functional activity of polyclonal immune sera [8]. We employed different analytical visualization methods to analyze the size and structure distribution of inactivated TBEV vaccine preparation.

Currently, cryo-EM is a popular method, which, unlike conventional TEM based on heavy metal compound contrasting, allows the objects under study to be vitrified in their native state. In addition, energy-filtered TEM combined with energy dispersive X-ray analysis (EDX) opens up new possibilities for analyzing the distribution of chemical elements in biological samples at the ultrastructural level. Recently, we developed a technique to study nucleic acid distribution by mapping phosphorus content in large DNA- and RNA-containing viruses at relatively low contents of the element of interest, based on scanning transmission electron microscopy and characteristic energy loss spectroscopy analysis (STEM-EELS) [9]. This technique allowed us to study the distribution of phosphorus in the capsid of DNA-containing bacteriophage EL (capsid diameter 145 nm), whose genome contains 211 kbp [10], which corresponds to ~422 thousand atoms of phosphorus, giving a prominent signal in the STEM-EELS.

In this work, the size and structure of purified inactivated TBEV virions were analyzed using four state-of-the-art physico-chemical methods. The dimensions of the virions measured using cryo-EM, negative contrast TEM (NC-TEM), atomic force microscopy (AFM), and nanoparticle tracking analysis (NTA) were compared. Additionally, a reconstruction of the electron density was obtained from the cryo-EM data, and the 5O6A model of the envelope protein was fitted into it. Finally, we recorded the phosphorus signal distribution within individual virions using STEM-EELS.

## 2. Materials and Methods

### 2.1. Obtaining Inactivated Virions of the TBEV

We used the Sofjin-Chumakov TBEV strain (Genbank KC806252): a prototype strain of the Far-Eastern subtype of the tick-borne encephalitis virus, which is used to produce commercial inactivated TBE vaccines (Chumakov FSC R&D IBP RAS (Institute of Poliomyelitis), Moscow, Russia) [11]. Reproduction of TBEV was performed in the primary cell culture of chicken embryo fibroblasts in Eagle’s MEM, with the addition of 2% fetal bovine serum, 2 mM L-glutamine, and 50 µg/mL gentamycin. The virus-containing liquid was collected 48–72 h after infection when a 75% cytopathic effect was reached. The virus titer was determined via the plaque-forming method and expressed as the logarithm of the number of plaque-forming units per ml (PFU/mL). Methylcellulose-coated titration was performed as described previously [12,13], the number of plaques in a cell was counted, and the TBEV titer was calculated according to the Reed and Muench method.

To inactivate TBEV, a formaldehyde solution was added to the virus-containing liquid to a final concentration of 0.02% and incubated at 32 °C for 72 h. After inactivation, a solution of protamine sulfate in a borate salt buffer solution (BSB, pH 8.5) was added to the virus-containing liquid to a final concentration of 50 mg/mL, incubated for 60 min at 4 °C, and centrifuged at 12,000 rpm for 30 min at 4 °C (Beckman Coulter, Brea, CA, USA). The supernatant was collected and centrifuged at 25,000 rpm for 3 h at 4 °C (Beckman Coulter, Brea, CA, USA). The resulting precipitate was resuspended in a TNE buffer solution (0.1 M Tris-HCl, 0.13 M NaCl, 1 mM EDTA, pH 7.8). The resulting solution was then layered onto a 15–60% weight/weight sucrose density step gradient and centrifuged at 35,000 rpm for 3.5 h at 4 °C (Beckman-Coulter, Brea, CA USA). Gradient fractions were collected, and target fractions were detected via enzyme immunoassays using a commercial kit Vector-VCE Antigen (Vector-Best, Russia) and through a 10% PAGE. The target fractions were combined, and the viral particles were resuspended in a TNE/5 buffer solution (20 mM Tris-HCl, 25 mM NaCl, 1 mM EDTA, pH 7.8) by centrifugation at 35,000 rpm for 3 h at 4 °C (Beckman-Coulter, Brea, CA USA). The resulting precipitate was dissolved in TNE/5 buffer. The samples were stored at 4 °C for no more than 7 days or at −70 °C.

### 2.2. Determination of the Hydrodynamic Size of Virions

The hydrodynamic size of the virions was determined via nanoparticle tracking analysis using a NanoSight NS500 (Malvern Instruments, Malvern, UK) instrument equipped with a 532 nm laser with a power of 80 mW. The virion-containing suspension was diluted with a TNE/5 buffer and measured according to the manufacturer’s instructions at 25 °C. For this purpose, the optimal concentration of virions was adjusted through successive dilution of the initial sample with a concentration of 2 × 10^13^ up to 10^8^–10^9^ particles/mL (20–40 particles per frame; the final dilution was 26.5 thousand times). The results of ten independent experiments (3722 tracks included in the distribution) were processed using the NanoSight NTA 3.4 software (Malvern Instruments, Malvern, UK).

### 2.3. Electron Microscopy

TBEV samples were applied on standard copper grids for transmission electron microscopy with a carbon coating (Ted Pella, Redding, CA, USA), and treated in a glow discharge atmosphere for 45 s in a Pelco EasyGlow apparatus (Ted Pella, Redding, CA, USA). For NC-TEM, the samples were negatively stained with either a 1% aqueous solution of uranyl acetate or a 2% aqueous solution of ammonium molybdate. Ammonium molybdate was used as a substitute for uranyl acetate in STEM-EELS experiments because the uranium M_4,5_ line precedes the phosphorus line in the characteristic electron energy loss spectra, and, thus, makes correct approximations of the background component difficult.

TEM images were obtained using a Jeol JEM-2100 electron microscope (JEOL, Japan) equipped with a LaB_6_ electron source and an accelerating voltage of 200 kV. To obtain characteristic electron energy loss spectra (EELS) and to build maps of the spatial distribution of elements, the dark-field scanning transmission electron microscopy (STEM) mode, in combination with a Gatan GIF Quantum ER spectrometer (Gatan, Pleasanton, CA, USA), was used. To reduce the effects of contamination and to stabilize the sample under the electron beam in STEM, a cooling holder JEOL 21090 (JEOL, Tokyo, Japan), supporting the sample at −180 °C, was used.

### 2.4. Spectroscopy of Characteristic Electron Energy Loss (EELS)

To obtain maps of the spatial distribution of the elements, the accumulation of spectra was synchronized with the recording of STEM images. Thus, each pixel of the STEM-image corresponded to the EELS spectrum taken from the corresponding area. A beam with a diameter of 5 nm was used to acquire spectra; the spectrometer collected the signal at a collection angle of 6 mrad. The spectra were recorded with an energy resolution of 0.25 eV/channel, with a full range of 2048 channels. The set of spectra corresponding to different pixels of the STEM image was aligned with the K-line of carbon at 283 eV. The background was extrapolated using a 109–128 eV window just before the phosphorus edge. No multiple scattering correction was applied. After background subtraction, the 132–155 eV range in the spectra was used to construct maps of the spatial distribution of phosphorus in the virions.

### 2.5. Cryo-Electron Microscopy

TBEV samples were applied to copper grids with a Lacey carbon substrate (Ted Pella, Redding, CA, USA), pre-treated with a glow discharge in a residual atmosphere (residual atmosphere discharge 0.26 mBar, 15 mA, 30 s) using a Pelco EasyGlow unit (Ted Pella, Redding, CA, USA). After application, the sample was incubated on a grid for 30 s, the excess liquid was removed with filter paper, and the sample was plunge-frozen in liquid ethane on a Leica EM GP2 device (Leica-microsystems, Wetzlar, Germany) with the following parameters: 4 °C, 95% relative humidity in an environmental chamber, blotting time ~6 sec, blotting on the carbon side only. The Jeol JEM-2100 TEM (JEOL, Tokyo, Japan) equipped with a Gatan Elsa cryotransfer holder (Gatan, Pleasanton, CA, USA), a DirectElectron DE-20 (Direct Electron, San Diego, CA, USA) direct electron detector, and SerialEM automated data acquisition software [14] were used to acquire images. Images were acquired with a pixel size of 1.4 Å, a total dose of 60 e/Å^2^, and a defocus of 1.5 μm.

### 2.6. Atomic Force Microscopy

To examine TBE virions with AFM, samples were applied to mica modified with nickel ions for 1 min in a 5 mM NiCl_2_ solution. After absorption, the sample was washed with water and dried with a stream of nitrogen.

Measurements were performed in a semicontact mode using a Solver PRO-M atomic force microscope (ZAO MDT Nanotechnology, Zelenograd, Russia) and NSG10 cantilevers (Kapella LLC, Zelenograd, Russia) with a nominal radius of curvature not exceeding 10 nm. The number of image points was 512 × 512 and the scanning frequency was 1.4 Hz.

### 2.7. Image Processing

The previously developed by us ScanEV program (https://bioeng.ru/scanev, accessed on 27 June 2022, [15]) was used to measure the size of virions in TEM images. Images obtained using cryo-EM were preliminarily processed: the number of pixels was reduced two-fold, then an averaging with a 3 × 3 pixel window was applied, and contrast was inverted. The images obtained in NS-TEM did not require any special preparation procedures.

The AFM images were processed using FemtoScan Online software (http://www.nanoscopy.net/en/Femtoscan-V.shtm, accessed on 6 September 2021, [16]). Upon measurements, we excluded the objects with less than 10 nm height from the sample.

### 2.8. Cryo-EM Reconstruction of the Tick-Borne Encephalitis Virus

The three-dimensional density map was reconstructed via single particle analysis in the Relion 3.1 program [17]. The first three-dimensional model was generated ab initio, and the reconstruction was performed with icosahedral symmetry applied. The final reconstruction was constructed using the projections from 2500 particles and yields an 8.0 Å resolution, estimated at 0.143 FSC. The docking of the envelope protein model into a three-dimensional map was performed using the *Fit in Map* routine in UCSF Chimera 1.15 [18].

## 3. Results and Discussion

### 3.1. Virion Size

**Figure 1:**
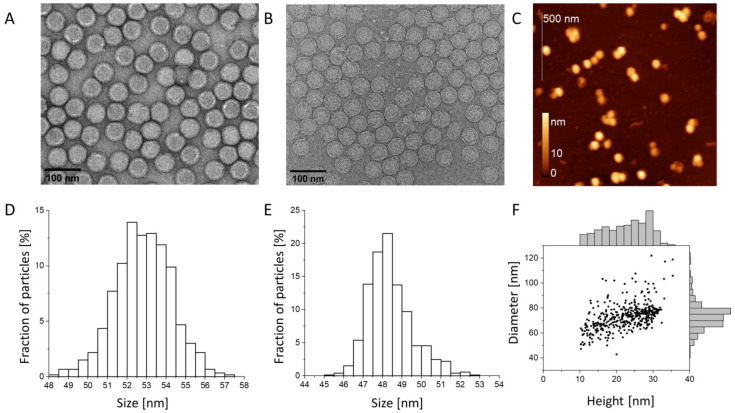
Tick-borne encephalitis virus (TBEV) virions visualization and size measurements. (**A**) Image obtained via NC-TEM, (**B**) Image obtained via cryo-EM; scale bars are 100 nm. (**C**) Image obtained via AFM; scale bar is 500 nm. (**D**–**F**) Particle size distributions obtained by methods presented in (**A**–**C**).

Measurements of virion diameters are important for controlling the homogeneity of the samples, building structural models, and for the applications of viruses in nanotechnology [19,20]. According to previous cryo-EM data, the virion diameter of TBEV is ~50 nm [2]. We compared virion diameters measured by different methods. Figure 1 shows images of the same inactivated TBEV sample obtained through NC-TEM, cryo-EM, and AFM (Figure 1A–C, respectively). All three methods show that most particles have a rounded shape; almost no damaged or immature particles [21] are observed. Such high homogeneity is, apparently, the result of the multi-step purification procedure described in the Section 2.

Virion size distributions obtained from the TEM data are shown in Figure 1D,E. The mean diameters were D_NC-TEM_ = 52.8 ± 1.4 nm and D_cryo_ = 48.4 ± 1.1 nm (mean ± standard deviation) using NC-TEM and cryo-EM, respectively. The difference can be explained by the deformation (flattening) of the virions caused by the adsorption onto the grid when preparing samples for NC-TEM. Thus, the samples are monodisperse: the ratio of the standard deviation of the diameter to the mean diameter was less than 3%.

Using the AFM images, we measured the diameters and the heights of individual virions (Figure 1E, the particles with height < 10 nm were excluded). The average diameter was D_AFM_ = 73 ± 11 nm, and the average height was h_AFM_ = 23 ± 6 nm. The relatively low height value is a consequence of the deformation of the virions due to the particle-substrate interaction and the compressive force of the cantilever tip. The diameter of the particles was noticeably higher than the expected value of ~50 nm, not only due to particle deformation but also to the broadening associated with the finite tip curvature radius. Similar deformations have been observed in previous TBEV studies [22,23].

**Figure 2:**
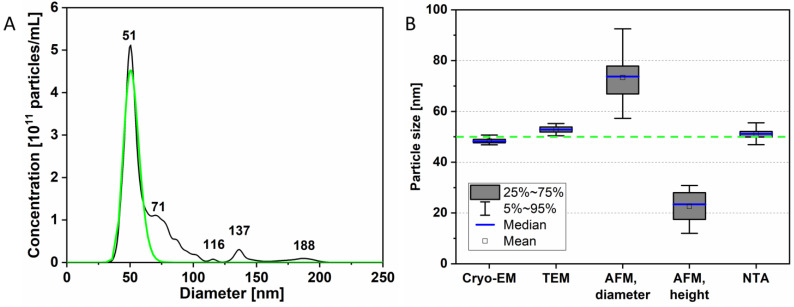
Comparison of TBEV particle sizes. (**A**) Distribution of the hydrodynamic size of TBEV obtained using the nanoparticle tracking analysis (NTA)—black line, and the approximation of the first peak using a log-normal function—green line. (**B**) Comparison of virion size data obtained within this study. The green dashed line in (**B**) shows the mean diameter of the virions according to literature data [2].

Nanoparticle tracking analysis (NTA) was used to measure the hydrodynamic diameter of the virions in the solution. The particle size distribution (Figure 2A) had a pronounced peak on the left (51 nm), as well as additional small peaks at 71, 116, 137, and 188 nm. These may indicate partial virion aggregation resulting from sample concentration and centrifugation during sample preparation. However, we cannot exclude that they may be an artifact of data processing [24].

Figure 2B compares the virion sizes obtained using different methods. For the NTA method, not the entire distribution was used, but an approximation of the main peak by a lognormal function (green curve in Figure 2A). This assumption can be applied to estimate the size of TBEV virions since the data obtained through TEM showed a high monodispersity of the samples. The results of measurements obtained using NC-TEM, cryo-EM, and NTA methods are in excellent agreement with each other and with literature data [1,2]. Slight discrepancies between them can be explained by the peculiarities of the measured quantities (virion diameter on the substrate for TEM, hydrodynamic diameter for NTA), instrumental errors, and specific aspects of data processing. AFM data, as indicated above, overestimated the diameter and underestimated the height; this is typical for small objects, including virions.

### 3.2. Analysis of TBEV Structure

**Figure 3:**
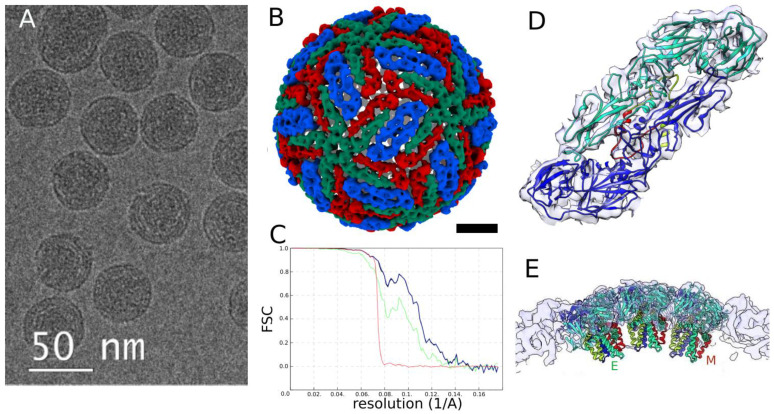
Cryo-EM study of inactivated TBEV. (**A**) Cryo-EM image of TBEV virions, scale bar is 50 nm; (**B**) Density map reconstructed from projections by single particle analysis, scale bar is 10 nm; (**C**) Fourier shell correlation plot: green—unmasked half-maps, blue—masked half-maps, red—phase randomized masked half-maps, black—corrected, (**D**) E protein dimer, two 5O6A models are docked into EM density, viewed from the surface of the virion, and (**E**) cross-section of the capsid shell. E proteins (marked E) are colored cyan and blue, and M proteins (marked M) are colored red and light green.

According to the TEM data, most virions were structurally intact (Figure 3A). Using single particle analysis, we obtained a three-dimensional reconstruction of TBEV with an average resolution of 8.0 Å according to FSC 0.143 criterion [25] (Figure 3B,C). The atomic model of TBEV envelope proteins (PDB: 5O6A) was docked to the obtained electron density map, which allowed us to identify the main structural features. The correlation coefficient between the electron density distribution and the model for the E protein was 0.75, indicating a good agreement between them.

The copies of E protein are organized in icosahedral symmetry with T = 3, each asymmetric subunit containing three E protein molecules. On the surface, E proteins formed the characteristic herringbone pattern (Figure 3D) of heterotetramers together with the M proteins. The E protein mediates the fusion of the cell and virion membranes [21] and influences the viral properties [26,27]. In flaviviruses, E proteins change their conformation upon pH decreases in the endosomes, so the membranotropic fragments, the fusion peptides, are exposed to the endosome membranes. Histidine residues play the role of the switch in this process [21].

In the obtained reconstruction, the signal-to-noise ratio allowed us to reliably fit the elements of the secondary structure located on the virion surface. However, the density map had a relatively low signal-to-noise ratio in those regions where the transmembrane alpha-helices of E and M proteins are located (Figure 3E). This may be due to the irregular arrangement of the transmembrane sites, which could break the symmetry, or as a result of virus particles’ inactivation by formaldehyde solution. The nucleocapsid of the virus does not have icosahedral symmetry; therefore, it is not represented in this reconstruction.

### 3.3. Phosphorus Mapping via STEM-EELS

**Figure 4:**
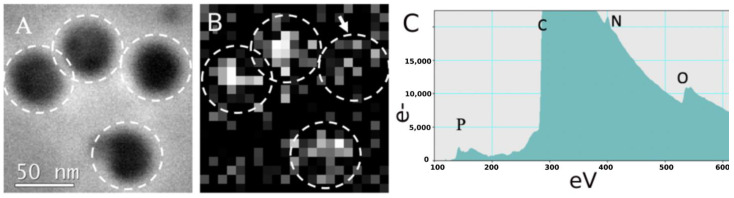
STEM-EELS analysis of TBEV. (**A**) STEM image of tick-borne encephalitis virus virions, (**B**) map of phosphorus EELS signal distribution in the same sample; white dotted lines indicate virion boundaries and arrow indicates virion with lower phosphorus signal, and (**C**) EELS spectrum from one representative virion. Letters indicate the spectrum edge positions for corresponding elements: P—phosphorus, C—carbon, N—nitrogen, and O—oxygen.

To reveal the distribution of RNA within virions, we constructed phosphorus distribution maps using STEM-EELS (Figure 4A). These allowed us to visualize the distribution of RNA in the nucleocapsids (Figure 4B). All of the virions used in this experiment yielded phosphorus signals (Figure 4C).

The phosphorus signal was maximal in the central region of the virion, but not in the periphery. This indirectly suggests that the source of the signal, in this case, was the phosphorus from the RNA, rather than from the lipid membrane. Similar phosphorus signal distribution was observed in our previous studies of SARS-CoV-2 virions [9] and EL bacteriophages [28]. Nevertheless, certain virions gave a considerably lower phosphorus signal than others (Figure 4B, arrow), suggesting that they lack RNA or the RNA is disordered.

The TBEV maturation process is quite complex and usually does not lead to the formation of perfectly identical particles. The selection of fractions according to their ELISA signal from the E proteins could not distinguish between empty particles, particles with partial genomes, and perfect particles. Even in early TEM studies, the mature, empty, and destroyed particles were co-sedimenting [29]. Cryo-EM studies of TBEV samples purified using simpler procedures compared to ours could also lead to less homogenous samples [1].

Another possible source of the differences in the distribution of the phosphorus signal between the virions could be attributed to an error in determining the background component in the spectrum. The background component depends on the local thickness of the sample expressed in mean free path electron lengths. The local thickness, in turn, varies due to the non-uniformity of the contrasting agent layer: ammonium molybdate. The construction of phosphorus elemental maps occurs at the detection limit for the EELS method, so the data have a low signal-to-noise ratio. However, even in this case, the EELS method confirms the presence of RNA in most of the particles studied, which confirms the overall homogeneity of the sample and perfectly agrees with the results described above (Figure 1).

## 4. Conclusions

In this work, we investigated the size and structure of inactivated TBEV virions using high-resolution microscopy and nanoparticle tracking analysis. TBEV itself is a relatively small virus, so it is a suitable model object for the development of novel structure research methods.

Measuring single virions, as well as other biological objects whose size is in the nanometer range, is a non-trivial task [30]. According to our NC-TEM, cryo-EM, and NTA data and the literature data [1,2], the size of a TBE virion is ~50 nm. The difference between the specific values obtained using several different methods can be explained by their specific features (sample preparation or data processing algorithms) or by instrumental errors. In the AFM measurements, the height of the particles above the substrate was smaller and the lateral size was larger than the characteristic value of ~50 nm.

The first three-dimensional reconstruction of an inactivated virion from the Sofjin-Chumakov TBEV strain (Far-Eastern subtype) was obtained here from cryo-EM data with a resolution of 8.0 Å (Figure 3). The docking of the 5O6A model obtained from a sample of native TBEV of the HYPR strain revealed the common orientation of the E protein. On the other hand, the density underneath the protein shell appeared to be smeared and did not exhibit any structural details, probably because of virus inactivation with formaldehyde.

The STEM-EELS analysis method we previously developed for virus studies showed phosphorus signals from individual TBEV virions. This confirms the presence of RNA, which could not be observed in the electron density distribution. Previously, the STEM-EELS method was applied for larger viruses (e.g., SARS-CoV-2 with a diameter of ~100 nm [9] or EL bacteriophage capsid with a diameter of 145 nm [28]), but its application to smaller virus particles described in this work allows us to estimate this method’s limits.

The techniques employed within this study can be useful for working with other enveloped viruses of small size, as well as for studying the properties of nanobiological objects.

## Data Availability

The datasets generated and analyzed during this study are available from the corresponding author on reasonable request.
